# Depressive symptoms and health service utilization among Chinese middle-aged and older adults: a national population-based longitudinal survey

**DOI:** 10.1186/s13033-020-00421-3

**Published:** 2021-01-06

**Authors:** Jing Guo, Dexia Kong, Liming Fang, Yingxue Zhu, Bo Zhang

**Affiliations:** 1 Center for Health Policy and Technology Evaluation, Department of Health Policy and Management, School of Public Health, Peking University, Beijing, P. R. China; 2 Institute for Health, Health Care Policy, and Aging Research, Rutgers University, Piscataway, NJ USA; 3 School of Insurance and Economics, University of International Business and Economics, Beijing, P. R. China; 4 School of Sociology, Huazhong University of Science and Technology, Wuhan, P. R. China; 5 Department of Neurology and ICCTR Biostatistics and Research Design Center, Boston Children’s Hospital and Harvard Medical School, Boston, MA USA

**Keywords:** Depressive symptoms, Outpatient care, Inpatient care, Urban–rural difference

## Abstract

**Objectives:**

This study aimed to (1) examine the cross-sectional and longitudinal relationships between depressive symptoms and health services utilization among Chinese middle-aged and older adults; and (2) evaluate whether there exists a rural–urban difference in such relationships.

**Methods:**

Data was obtained from China Health and Retirement Longitudinal Study (CHARLS) in 2013 and 2015, a nationally representative survey of 13,551 adults aged 45 years and above in China.

**Results:**

Depressive symptoms were positively associated with a greater likelihood of outpatient and inpatient health services utilization. This association was consistent across rural and urban settings, indicating the robustness of such findings across geographic areas.

**Conclusions:**

Findings indicate that depressive symptoms are significantly associated with both in-patient and out-patient health service utilization among Chinese adults. Screening for depressive symptoms needs to be incorporated in these care settings in China.

## Introduction

The prevalence of depressive symptoms has been steadily increasing in recent years due to improved identification and reporting of cases and to cultural and social transitions. [1]. A recent meta-analytic review suggested that approximately 17–24% of adults in China experience depressive symptoms [2, 3]. It has been well documented that depressive symptoms are associated with disability and mortality [4, 5]. However, according to the results of mental health surveys conducted by the World Health Organization (WHO) in 17 countries, middle-aged and older adults were less likely to use mental health services, and unmet needs for mental health treatment were pervasive in developing countries [6]. Thus, elucidating the relationship between mental health service utilization and depressive symptoms among Chinese middle-aged and older adults may increase public awareness of mental disorders in this cohort.

An emerging body of literature in Western countries has examined the relationship between depressive symptoms and utilization of health services worldwide. Various studies have indicated that depressive symptoms are related to health service use [7, 8]. A previous population-based study in the United States reported that Chinese immigrants with depressive symptoms were nearly twice as likely to utilize acute care (i.e. hospitalization and emergency department visits) than their counterparts without depressive symptoms [9]. However, whether such a relationship holds true for middle-aged and older adults in China remains unclear. Additionally, previous studies used cross-sectional designs, which make causal inferences difficult to determine.

An improved understanding of the relationship between depressive symptoms and health services utilization in Chinese middle-aged and older adults is therefore warranted, for several reasons. First, mental health service infrastructure in mainland China is less developed than in many Western countries [10]. Second, among Chinese adults depression and depressive symptoms are often expressed in somatic symptoms, such as insomnia and chest pain [11]. As a result, Chinese middle-aged and older adults in mainland China are likely to have help-seeking behaviors that are culturally informed and distinct from their counterparts in, for example, the United States, where the mental health system is more established [12].

Furthermore, also unique to mainland China’s middle-aged and older population are the salient mental health disparities between its urban and rural communities. Chinese adults in rural areas have consistently reported much higher rates of mental health disorders than their urban counterparts [13], with studies among rural Chinese adults reporting a prevalence of mental disorders of up to 60% [14]. Nevertheless, compared to rural areas in China, urban Chinese areas tend to have more mental health resources and services [15]. These rural–urban disparities in mental health and related health-seeking behaviors remain largely unexplored [3].

To address these significant knowledge gaps, the present study aimed to 1) examine the cross-sectional and longitudinal relationships between depressive symptoms and health services utilization among Chinese middle-aged and older adults, and 2) evaluate whether there exists a rural–urban difference in such relationships.

## Design and methods

### Data source, procedure and participants

This paper used data from China Health and Retirement Longitudinal Study (CHARLS), a nationally representative survey of more than 17,000 Chinese adults aged 45 years and above from rural and urban China. Since 2011, CHARLS has collected information on respondents’ socio- demographic characteristics, retirement and economic well-being, health-related behaviors, and health services utilization every two years. Participants were selected using a multistage, stratified, cluster sampling strategy. First, 150 counties in China were randomly selected, according to geographical location and relative level of socioeconomic development. Second, in each county, three major villages were selected, with probability proportional to population size. Third, all households in each selected unit were included in the sampling frame, and 24 households were randomly drawn from all the households. Finally, within each selected household, one member over 45 years old was selected as a subject of the survey. The baseline survey of the CHARLS involved 17,708 respondents, in 2011. Detailed information on sampling has been published elsewhere [16]. The analytic sample in this present study included a total of 13,551 individuals who completed the survey in both 2013 and 2015 (representing 77% of the baseline population).

### Measurement

*Participant’s utilization of inpatient and outpatient care services* in 2013 and 2015 were dependent variables in this study. Inpatient care service use was assessed by asking “Have you received inpatient care in the past year?” Participants responded yes/no. Outpatient service use was measured by asking “In the last month have you visited a public hospital, private hospital, public health center, clinic, or health worker’s or doctor’s practice, or been visited by a health worker or for outpatient care?” Participants responded yes/no. The same questions were asked in both waves (2013 and 2015).

*Depressive symptoms* were assessed using the 10-item Center for Epidemiological Studies Depression Scale (CESD-10), a widely-used instrument of depressive symptoms in community samples [17]. Participants indicated the extent to which they experienced ten symptoms during the last week, such as sleep problems and feeling that everything was an effort, on a four-point Likert scale: 0 (less than 1 day), 1 (1–2 days), 2 (3–4 days), or 3 (5–7 days). Total score ranged from 0 to 30, with higher scores indicating greater levels of depressive symptoms. A previous study reported satisfactory psychometric properties of CESD-10 among Chinese adults [18]. A cut-off point of 12 was adopted in the present study to define the presence of clinically significant depressive symptoms because a previous study reported that a cut-off of 12 has a specificity of 0.55 and a sensitivity of 0.76 to detect depression [19]. The Cronbach's alpha of the scale was 0.80 in the study sample.

*Significant covariates* reported in previous studies were included as covariates [1, 2, 6]. Sociodemographic variables included age (in years), gender (male/female), marital status (married/not married), education (in years), self-reported health (good/fair/poor), chronic disease (yes/no), smoking history (yes/no), family monthly income, residential type (urban/rural) and medical insurance (yes/no). Region (eastern region/central region/western region) and hukou status (agriculture/non-agricultural) were added to capture socioeconomic characteristics that are unique to Chinese middle-aged and older adults.

### Statistical analyses

Data were analyzed using Stata Version 14. Descriptive statistics were calculated to describe the sociodemographic and health characteristics of the sample. Chi-square tests (for categorical variables) and t-tests (for continuous variables) were used to determine if there existed any rural/urban difference in participants’ demographic and health characteristics. Meantime, these methods were also conducted to compare depressive symptoms and socio-demographic characteristics by inpatient and outpatient care utilization. Multivariate logistic regression analyses were conducted using 2013 data to assess the cross-sectional relationship between depressive symptoms and health services utilization in the whole sample, and urban and rural subsamples respectively, controlling for covariates. To examine the longitudinal relationship, multivariate logistic regression analyses were conducted using self-reported depressive symptoms in 2013 as the independent variable and health service use in 2015 as the dependent variable. Considering the large sample size (> 10,000), a conservative p value (i.e. *p* < 0.01) was adopted to establish statistical significance in the present study [20, 21].

## Results

### Descriptive statistics

Descriptive analyses for the participants in 2013 are presented in Table 1. Of the 13,551 participants, 51.62% were female, 89.92% were married, 79.63% were registered as agricultural hukou, and 44.59% (N = 6,042) had a history of smoking. In addition, about 62.73% of participants were living in rural areas. Regional distribution of the sample was 38.64% east, 25.43% central, and 35.93% west. The mean age was 59.30 years (SD = 9.14), and the average years of education was 5.39.

**Table 1:** Health-care service utilization and socio-demographic characteristics of the sample

Variables	Total frequency (%)	Urban (%)	Rural (%)	P-value
*Inpatient care in 2013*				0.006
No	11,867 (87.57)	4372 (86.56)	7495 (88.18)	
Yes	1684 (12.43)	679 (13.44)	1005 (11.82)	
*Outpatient care in 2013*				0.703
No	10,533 (77.73)	3935 (77.91)	6598 (77.62)	
Yes	3018 (22.27)	1116 (22.09)	1902 (22.38)	
*Inpatient care in 2015*				0.011
No	11,637 (85.88)	4288 (84.89)	7349 (86.46)	
Yes	1914 (14.12)	763 (15.11)	1151 (13.54)	
*Outpatient care in 2015*				0.226
No	10,754 (79.36)	4036 (79.90)	6718 (79.04)	
Yes	2797 (20.64)	1015 (20.10)	1782 (20.96)	
*Gender*				0.028
Male	6556 (48.38)	2382 (47.16)	4174 (49.11)	
Female	6995 (51.62)	2669 (52.84)	4326 (50.89)	
*Marital status*				0.518
Unmarried	1379 (10.18)	503 (9.96)	876 (10.31)	
Married	12,172 (89.92)	4548 (90.04)	7624 (89.69)	
*Hukou status*				< 0.001
Non-agricultural hukou	2761 (20.37)	2374 (47.00)	387 (4.55)	
Agricultural hukou	10,790 (79.63)	2677 (53.00)	8113 (95.45)	
*Smoking history*				< 0.001
No	7509 (55.41)	2888 (57.18)	4621 (54.36)	
Yes	6042 (44.59)	2163 (42.82)	3879 (45.64)	
*Medical insurance*				0.507
No	441 (3.25)	171 (3.39)	270 (5.35)	
Yes	13,110 (96.75)	4880 (96.61)	8230 (94.65)	
*Self-report health*				< 0.001
Good	3182 (23.48)	1296 (25.66)	1886 (22.19)	
Fair	7196 (53.10)	2835 (56.13)	4361 (51.31)	
Poor	3173 (23.42)	920 (18.21)	2253 (26.50)	
*Chronic disease*				0.730
No	3813 (28.14)	1430(28.31)	2383 (28.04)	
Yes	9738 (71.86)	3621(71.69)	6117 (71.96)	
*Current living place*				
Rural	8500 (62.73)	–	–	
Urban	5051 (37.27)	–	–	
*Region*				< 0.001
East	5236 (38.64)	2166 (42.88)	3070 (36.12)	
Central	3446 (25.43)	1262 (24.99)	2184 (25.69)	
West	4869 (35.93)	1623 (32,13)	3246 (38.19)	
*Depression*				< 0.001
No	10,354 (76.41)	4120 (81.57)	6234 (73.34)	
Yes	3197 (23.59)	931 (18.43)	2266 (26.66)	
*Variable*	Mean (Std.)			
Age	59.30 (9.14)	59.44 (9.22)	59.21 (9.09)	0.147
Education years	5.39 (4.15)	6.75 (4.25)	4.59 (3.88)	< 0.001
Income	4.29 (1.04)	4.63 (1.01)	4.10 (1.01)	< 0.001
N	13,551	8500	5051	

“1.000” was the result of keeping 3 decimal places

Income was logarithm

*Std.* Standard deviation;

In terms of self-report health status, 23.48% of participants reported that their health status was good, while the rates for fair and poor status were 53.10% and 23.42%, respectively. About 27.88% of the respondents were found to have depressive symptoms using 10 as the cut-off point. In addition, most participants (71.86%) indicated that they had chronic disease. Overall, compared to the Chinese urban population, rural respondents were more likely to receive outpatient care and were more likely to have depressive symptoms (22.38% and 26.66%, respectively).

In terms of health care utilization, in 2013, the proportion of respondents who received inpatient care and outpatient care were 12.43% and 22.27%, while in 2015 the rate of inpatient care and outpatient care were 14.12% and 20.64%, respectively.

### Bivariate correlation analyses

Table 2 shows the results of comparison of depressive symptoms and socio-demographic characteristics by inpatient and outpatient care utilization in 2013. The following characteristics were associated with greater likelihood of inpatient care: unmarried, non-agricultural hukou, presence of medical insurance, poor self-report health status, presence of chronic disease, urban residence, west region, and depressive symptoms. Similarly, the following characteristics were associated with greater likelihood of outpatient care: unmarried, presence of medical insurance, poor self-report health status, presence of chronic disease, west region, oldere age, higher education, and depressive symptoms.

**Table 2:** Comparison of depressive symptoms and socio-demographic characteristics by inpatient and outpatient care utilization in 2013 (N = 13,551)

Variables	Inpatient			Outpatient		
	No	Yes	P-value	No	Yes	P-value
*Gender*			0.236			< 0.000
Male	6103 (87.25)	892 (12.75)		5237 (74.87)	1758 (25.13)	
Female	5764 (87.92)	792 (12.08)		5296 (80.78)	1260 (19.22)	
*Marital status*			0.011			0.001
Unmarried	1178 (85.42)	201 (14.58)		1022 (74.11)	357 (25.89)	
Married	10,689 (87.82)	1483 (12.18)		9511 (78.14)	2661 (21.86)	
*Hukou status*			0.004			0.445
Non-agricultural hukou	2373 (85.95)	388 (14.05)		2161 (78.27)	600 (21.73)	
Agricultural hukou	9494 (87.99)	1296 (12.01)		8372 (77.59)	2418 (22.41)	
*Smoking history*			0.377			< 0.000
No	6559 (87.35)	950 (12.65)		5673 (75.55)	1836 (24.45)	
Yes	5308 (87.85)	734 (12.15)		4860 (80.44)	1182 (19.56)	
*Medical insurance*			< 0.000			0.045
No	410 (92.97)	31 (7.03)		360 (81.63)	81 (18.37)	
Yes	11,457 (87.39)	1653 (12.61)		10,173 (77.60)	2937 (22.40)	
*Self-report health*			< 0.000			< 0.000
Good	2992 (94.03)	190 (5.97)		2849 (89.53)	333 (10.47)	
Fair	6485 (90.12)	711 (9.88)		5720 (79.49)	1476 (20.51)	
Poor	2390 (75.32)	783 (24.68)		1964 (61.90)	1209 (38.10)	
*Chronic disease*			< 0.000			< 0.000
No	3605 (95.54)	208 (5.46)		3374 (88.49)	439 (11.51)	
Yes	8262 (84.84)	1476 (15.16)		7159 (73.52)	2579 (26.48)	
*Current living place*			0.006			0.703
Rural	7495 (88.18)	1005 (11.82)		6598 (77.62)	1902 (22.38)	
Urban	4372 (86.56)	679 (13.44)		3935 (77.91)	1116 (22.09)	
*Region*			< 0.000			< 0.000
East	4729 (90.32)	507 (9.68)		4250 (81.17)	986 (18.83)	
Central	2983 (86.56)	463 (13.44)		2624 (76.15)	822 (23.85)	
West	4155 (85.34)	714 (14.66)		3659 (75.15)	1210 (24.85)	
*Depression*			< 0.000			< 0.000
No	9277 (89.60)	978 (10.40)		8380 (80.93)	1974 (19.07)	
Yes	3072 (81.01)	706 (18.99)		2153 (67.34)	1044 (32.66)	
*Variable*	Mean (Standard Deviation)	Mean (Standard Deviation)		Mean (Standard Deviation)	Mean (Standard Deviation)	
Age	59.00 (9.07)	61.40 (9.39)	< 0.000	59.14 (9.09)	59.84 (9.29)	< 0.000
Education (years)	5.44 (4.16)	5.10 (4.10)	0.002	5.48 (4.15)	5.11 (4.17)	< 0.000
Income	4.29 (1.04)	4.31 (1.02)	0.400	4.28 (1.04)	4.35 (1.04)	< 0.001

### Binary logistic regression analyses

Logistic analyses were used to assess the relationship between depressive symptoms and health services utilization among Chinese middle-aged and older adults in 2013. For participants’ health services utilization in 2013 (Table 3), after adjusting for sociodemographic and other covariates, depressive symptoms were significantly associated with inpatient care and outpatient care. Compared to rural residents, urban elderly adults were more likely to seek inpatient care.

**Table 3:** Logistic regression analysis for the relationship between depress symptoms and health care utilization among Chinese middle aged and older adults in 2013

Variables	Model 1	Model 2	Model 3	Model 4	Model 5	Model 6
	Inpatient care in 2013 (Full model)	Outpatient care in 2013 (Full model)	Inpatient care in 2013 (Rural)	Outpatient care in 2013 (Rural)	Inpatient care in 2013 (Urban)	Outpatient care in 2013 (Urban)
	OR [95%CI]	OR [95%CI]	OR [95%CI]	OR [95%CI]	OR [95%CI]	OR [95%CI]
*Depression (no)*						
Yes	1.349*** [1.194,1.524]	1.366*** [1.238,1.508]	1.276** [1.096,1.486]	1.400*** [1.241,1.578]	1.496*** [1.219,1.835]	1.287** [1.082,1.531]
Age	1.024*** [1.018,1.031]	1.005^+^ [1.000,1.010]	1.018*** [1.009,1.026]	1.007* [1.000,1.013]	1.035*** [1.024,1.045]	1.002 [0.993,1.010]
Education	1.005 [0.989,1.021]	1.007 [0.994,1.019]	1.012 [0.991,1.034]	1.008 [0.992,1.025]	0.999 [0.976,1.024]	1.005 [0.985,1.025]
*Gender (female)*						
Male	1.107 [0.941,1.304]	0.877* [0.769,1.000]	0.971 [0.781,1.207]	0.846^+^ [0.713,1.004]	1.301* [1.013,1.669]	0.934 [0.759,1.149]
*Marital status (unmarried and others)*						
Married	1.077 [0.906,1.281]	0.929 [0.807,1.069]	0.970 [0.780,1.206]	0.965 [0.809,1.152]	1.241 [0.929,1.660]	0.867 [0.687,1.093]
*Hukou status (non-agricultural hukou)*						
Agricultural hukou	0.873^+^ [0.745, 1.023]	1.023 [0.898,1.166]	0.820 [0.599,1.124]	1.085 [0.828,1.424]	0.853 [0.703,1.035]	1.009 [0.862,1.181]
*Smoking history (no)*						
Yes	0.929 [0.794,1.087]	0.873* [0.769,0.991]	0.990 [0.805,1.217]	0.890 [0.755,1.048]	0.853 [0.669,1.087]	0.845 [0.689,1.036]
*Medical insurance (no)*						
Yes	1.819** [1.245,2.657]	1.196 [0.927,1.544]	1.522^+^ [0.958,2.418]	1.174 [0.847,1.626]	2.430** [1.252,4.714]	1.214 [0.805,1.833]
*Self-report health (good)*						
Fair	1.422*** [1.199,1.686]	1.824*** [1.600,2.078]	1.182 [0.946,1.477]	1.829*** [1.538,2.175]	1.807*** [1.385,2.358]	1.828*** [1.498,2.231]
Poor	3.639*** [3.033,4.366]	3.627*** [3.136,4.195]	2.989*** [2.371,3.769]	3.595*** [2.981,4.336]	4.848*** [3.615,6.502]	3.720*** [2.943,4.702]
*Chronic disease (no)*						
Yes	1.955*** [1.668,2.291]	1.897*** [1.690,2.130]	2.150*** [1.743,2.653]	1.909***[1.647,2.213]	1.703*** [1.335,2.172]	1.889*** [1.568,2.276]
*Current living place (rural)*						
Urban	1.244*** [1.094,1.414]	1.044 [0.941,1.157]				
*Region (east)*						
Central	1.323*** [1.152,1.520]	1.224*** [1.098,1.365]	1.322** [1.102,1.587]	1.442*** [1.253,1.658]	1.330** [1.072,1.649]	0.952 [0.799,1.134]
West	1.416*** [1.247,1.607]	1.230*** [1.113,1.359]	1.393*** [1.181,1.643]	1.341*** [1.180,1.526]	1.466*** [1.200,1.791]	1.096 [0.933,1.288]
Income	1.074* [1.016,1.135]	1.133*** [1.084,1.184]	1.102** [1.027,1.182]	1.121*** [1.061,1.185]	1.029 [0.941,1.126]	1.148*** [1.065,1.238]
Constant	0.003*** [0.002,0.006]	0.029*** [0.017,0.050]	0.006*** [0.002,0.016]	0.023*** [0.011,0.048]	0.001*** [0.000,0.005]	0.041*** [0.017,0.095]
R2	0.080	0.070	0.074	0.077	0.094	0.061
N	13,551	13,551	8500	8500	5051	5051

Parentheses was reference group and 95% confidence interval of odds ratio

“1.000/0.000” was the result of keeping 3 decimal places

*OR* odds ratio

***p < 0.001, **p < 0.01, *p < 0.05, ^+^p < 0.1

Within the rural sample, depressive symptoms were significantly associated with inpatient care (OR 1.276, 95% CI 1.096–1.486) and outpatient care (OR 1.400, 95% CI 1.241–1.578). In the urban sample, depressive symptoms were significantly associated with inpatient care (OR 1.496, 95% CI 1.219–1.835) and outpatient care (OR 1.287, 95% CI 1.082–1.531).

The relationship between depressive symptoms and participants’ health services utilization was further verified by full models. Table 4 shows the longitudinal relationship between depressive symptoms in 2013 and health care utilization in 2015. In the whole sample, depressive symptoms were significantly associated with inpatient care (OR 1.209, 95% CI 1.075–1.360) and outpatient care (OR 1.394, 95% CI 1.261–1.542). Adopting a conservative p value of 0.01, this relationship persists across other sub-models except Model 5. The relationship between depressive symptoms and participants’ inpatient care utilization was weak in the rural sample (OR 1.219, 95% CI 1.055–1.408) and in the urban sample (OR 1.190, 95% CI 0.972–1.458).

**Table 4:** Logistic regression analysis for the relationship between depress symptoms and health care utilization among Chinese older adults in 2015

Variables	Model1	Model2	Model3	Model4	Model5	Model6
	Inpatient care in 2015 (Full model)	Outpatient care in 2015 (Full model)	Inpatient care in 2015 (Rural)	Outpatient care in 2015 (Rural)	Inpatient care in 2015 (Urban)	Outpatient care in 2015 (Urban)
	OR [95%CI]	OR [95%CI]	OR [95%CI]	OR [95%CI]	OR [95%CI]	OR [95%CI]
*Depression (no)*						
Yes	1.209** [1.075,1.360]	1.394*** [1.261,1.542]	1.219** [1.055,1.408]	1.399*** [1.238,1.581]	1.190^+^ [0.972,1.458]	1.369*** [1.146,1.636]
Age	1.032*** [1.026,1.038]	1.004 [0.999,1.009]	1.027*** [1.019,1.035]	1.005 [0.998,1.012]	1.040*** [1.030,1.050]	1.001 [0.993,1.010]
Education	1.0004 [0.9857,1.0152]	1.016* [1.003,1.028]	0.999 [0.979,1.019]	1.010 [0.994,1.026]	1.003 [0.981,1.026]	1.028** [1.007,1.049]
*Gender (female)*						
Male	1.083 [0.930,1.261]	0.883^+^ [0.774,1.008]	1.134 [0.927,1.388]	0.837* [0.705,0.993]	1.003 [0.793,1.268]	0.964 [0.780,1.191]
*Marital status (unmarried and others)*						
Married	0.934 [0.798,1.092]	0.995 [0.862,1.149]	0.788* [0.649,0.956]	1.002 [0.837,1.199]	1.250 [0.954,1.636]	0.955 [0.749,1.217]
*Hukou status (non-agricultural hukou)*						
Agricultural hukou	0.823* [0.709,0.955]	0.979 [0.858,1.117]	0.842 [0.626,1.133]	0.762* [0.594,0.979]	0.819* [0.684,0.982]	1.091 [0.929,1.281]
*Smoking history (no)*						
Yes	0.909 [0.786,1.053]	0.909 [0.800,1.033]	0.845^+^ [0.698,1.023]	0.894 [0.759,1.052]	1.013 [0.807,1.272]	0.926 [0.753,1.139]
*Medical insurance (no)*						
Yes	1.260 [0.924,1.718]	1.157 [0.896,1.493]	1.219 [0.819,1.812]	1.072 [0.781,1.472]	1.295 [0.788,2.130]	1.327 [0.861,2.045]
*Self-report health (good)*						
Fair	1.384*** [1.193,1.606]	1.389*** [1.230,1.568]	1.458*** [1.189,1.788]	1.264** [1.082,1.476]	1.303* [1.047,1.622]	1.605*** [1.320,1.952]
Poor	2.343*** [1.986,2.765]	1.905*** [1.655,2.192]	2.416*** [1.940,3.008]	1.633*** [1.370,1.946]	2.267*** [1.751,2.937]	2.472*** [1.951,3.133]
*Chronic (No)*						
Yes	1.982*** [1.718,2.287]	1.725*** [1.542,1.931]	2.115*** [1.751,2.555]	1.828*** [1.584,2.110]	1.807*** [1.450,2.251]	1.579*** [1.315,1.897]
*Current living place (rural)*						
Urban	1.128^+^ [0.999,1.274]	0.945 [0.851,1.049]				
*Region (east)*						
Central	1.101 [0.967,1.254]	1.148* [1.029,1.280]	1.090 [0.919,1.293]	1.216** [1.057,1.399]	1.125 [0.920,1.376]	1.060 [0.890,1.264]
West	1.264*** [1.125,1.421]	1.148** [1.039,1.269]	1.241** [1.066,1.444]	1.259*** [1.108,1.430]	1.296** [1.077,1.560]	0.979 [0.829,1.156]
Income	1.038 [0.986,1.091]	1.070** [1.024,1.119]	1.044 [0.979,1.113]	1.063* [1.006,1.123]	1.024 [0.942,1.112]	1.078^+^ [1.000,1.163]
Constant	0.006*** [0.003,0.012]	0.056*** [0.032,0.095]	0.009*** [0.004,0.022]	0.075*** [0.037,0.152]	0.004*** [0.001,0.010]	0.045*** [0.019,0.107]
R2	0.056	0.033	0.057	0.035	0.055	0.034
N	13,551	13,551	8500	8500	5051	5051

Parentheses was reference group and 95% confidence interval of odds ratio;

“1.000” was the results of keeping 3 decimal places

***p < 0.001, **p < 0.01, *p < 0.05, ^+^p < 0.1

*OR* odds ratio

## Discussion

Consistent with an emerging body of literature on the relationship between depressive symptoms in adults and their utilization of health services, our findings show a consistent association between depressive symptoms and inpatient and outpatient service use among Chinese middle-aged and older adults. Unlike previous literature that predominantly focused on health services utilization in acute care settings, the present study investigated Chinese adults’ health services utilization in both inpatient and outpatient settings.

Depressive symptoms were positively associated with a greater likelihood of outpatient health services utilization. There is a relatively low awareness of mental disorders in mainland China, and middle-aged and older adults who experience depression are likely to present with somatic symptoms [6]. As a result, this cohort of adults may seek care from physicians instead of mental health professionals. It has been well documented that depressive symptoms and depression among middle-aged and older adults are largely underdiagnosed and under‐treated in primary care settings [22]. Prior studies have found that an elderly person whose depression is undiagnosed may unnecessarily use healthcare services, such as outpatient visits to primary care physicians, emergency departments, and urgent care centers, thereby increasing medical resource utilization [8]. Our findings strongly suggest that appropriate screening and treatment of depressive symptoms need to be incorporated in outpatient clinical encounters, thereby preventing the onset of depression and associated physical diseases, and improving the quality of life among elderly persons in China.

Furthermore, our findings suggest that depressive symptoms in Chinese middle-aged and older adults’ also led to utilization of inpatient health services. It can be explained by that this cohort experiences chronic medical conditions are often comorbid with depressive symptoms, such as diabetes and heart disease [23]. These patients may misinterpret somatic symptoms of depression as worsening physical conditions. This possibly explains the increased inpatient service use because, as their depression further develops, they start to think outpatient services are simply not enough. Depressed adults are less likely to adapt well to their physical conditions and often will have increased burden of disease due to low motivation to manage their physical conditions [24]. Consequently, as their physical health further deteriorates, their inpatient services utilization will increase. It is also possible that depressive symptoms may be an adjustment disorder to poor physical status itself. A final explanation is due to over-investigation and unnecessary referral to hospitalization often known as “a diagnostic stumbling block” in the mainland China healthcare system. One distinct characteristic of mainland China’s healthcare system is that most patients seek primary and secondary healthcare services in tertiary hospitals. Somatic symptoms and hypochondriasis due to depression may be misdiagnosed as worse physical conditions, resulting in inappropriate treatment and referral to inpatient care.

Importantly, our data indicate that the relationships between depressive symptoms and health service use were consistent across rural and urban settings, indicating the robustness of such findings across geographic areas. Moreover, our findings suggest that older Chinese adults in urban areas had a higher rate of utilization of health service in general than those in rural areas. Many factors play an important role in explaining this result. First, this may partly be attributable to the urban–rural inequality in health care in China. Ma et al. [25] suggested large and long-term urban–rural disparities in health and health care in China due to urban–rural dualistic systems. Moreover, utilization of health care is related to income [26]. The expense of health services, especially inpatient service–the greater part of which is not reimbursed by the Chinese public health system–might be responsible for the lower rate of health services utilization among rural residents. Other studies have shown that depression literacy or cultural traditions can affect utilization of health services among older people with depression. For example, subjects who lived outside major cities regarded counselors or psychologists as less helpful than those who lived in major metropolitan cities [27].

### Implications for public health policy

Based on these findings, a program of early screening and intervention will improve primary care providers’ recognition of depression symptoms and make specialized mental health treatments available. Such a program may reduce the incidence of chronic disease and physical disease among elderly adults. In 2017, China implemented a public health policy for free adult preventive care services among adults aged 65 and over; however, these services focus mainly on physical examinations. This program should be extended to include mental status examinations by adding a CES-D survey as an initial attempt to detect depressive symptoms.

Secondly, it is necessary to provide a well-established set of interventions and routine medical checkups to high risk groups among the elderly, especially rural people. Thirdly, in view of the various comorbid chronic conditions and complex care needs of elderly people, comprehensive geriatric assessments, such as outpatient geriatric evaluation and management, is a possible approach to reduce the burden of morbidity and medical utilization. Such comprehensive management would enable better screening for depression among elderly patients with chronic disease.

## Strengths and limitations

Several limitations should be considered when interpreting our findings. First, CES-D is a depressive symptom screening tool rather than a clinical diagnostic measure. Particularly, since CES-D includes somatic symptoms (poor appetite, poor sleep) which may be influenced by physical status, future studies need to validate the findings using clinical diagnostic measures of depressive symptoms. Second, both depressive symptoms and health care utilization were self-reported and may be subject to recall bias. Considering the stigma associated with mental illness among Chinese populations, depressive symptoms may be underreported. Lastly, other potential variables that may predict health service use, such as health beliefs and health literacy, were not included in the present study.

## Conclusion

The study findings suggest that depressive symptoms are significantly associated with both in-patient and out-patient health service utilization in Chinese middle-aged and older adults. Screening for depressive symptoms needs to be incorporated in these care settings in China. Appropriate treatment of depressive symptoms and timely referrals to mental health services may optimize utilization of health services among Chinese middle-aged and older adults.

## Data Availability

The datasets used and/or analyzed during the current study are available from the corresponding author on reasonable request.

## Abbreviations

CHARLS: China Health and Retirement Longitudinal Study
WHO: World Health Organization
US: United States
CESD-10: 10-Item Center for Epidemiological Studies Depression Scale
N: Number
Std.: Standard deviation
OR: Odds Ratio
95% CI: 95% Confidence interval
